# Unusual Force Constants Guided Distortion-Triggered Loss of Long-Range Order in Phase Change Materials

**DOI:** 10.3390/ma14133514

**Published:** 2021-06-24

**Authors:** Jiong Wang, Dongyu Cui, Yi Kong, Luming Shen

**Affiliations:** 1 Powder Metallurgy Research Institute, Central South University, Changsha 410083, China; wangjionga@csu.edu.cn (J.W.); dongyucui@csu.edu.cn (D.C.); 2 School of Civil Engineering, The University of Sydney, Sydney, NSW 2006, Australia; luming.shen@sydney.edu.au

**Keywords:** unusual force constant, phase change materials, GeTe, Sb_2_Te_3_

## Abstract

Unusual force constants originating from the local charge distribution in crystalline GeTe and Sb_2_Te_3_ are observed by using the first-principles calculations. The calculated stretching force constants of the second nearest-neighbor Sb-Te and Ge-Te bonds are 0.372 and −0.085 eV/Å^2^, respectively, which are much lower than 1.933 eV/Å^2^ of the first nearest-neighbor bonds although their lengths are only 0.17 Å and 0.33 Å longer as compared to the corresponding first nearest-neighbor bonds. Moreover, the bending force constants of the first and second nearest-neighbor Ge-Ge and Sb-Sb bonds exhibit large negative values. Our first-principles molecular dynamic simulations also reveal the possible amorphization of Sb_2_Te_3_ through local distortions of the bonds with weak and strong force constants, while the crystalline structure remains by the X-ray diffraction simulation. By identifying the low or negative force constants, these weak atomic interactions are found to be responsible for triggering the collapse of the long-range order. This finding can be utilized to guide the design of functional components and devices based on phase change materials with lower energy consumption.

## 1. Introduction

Phase change materials can exist in at least two different phases, such as a crystalline phase and an amorphous phase, featuring rapid and reversible switching between phases with large property contrasts. The most widely used phase change materials for rewritable optical disks are GeTe-Sb_2_Te_3_ pseudobinary compounds [1,2,3]. The unique property of GeTe-Sb_2_Te_3_ also makes it an excellent candidate for various applications in computer science, especially in the field of non-volatile computer memory [4,5]. Important progress has been achieved in the past 20 years to develop new phase change materials and understand their phase change mechanisms [6,7,8,9,10,11,12,13,14,15]. It has been known that local interactions between atoms play an important role in phase changes [4,16]. For example, a recent study reveals that local distortions in crystalline can trigger the collapse of long-range order, leading to the formation of the amorphous phase without going through the liquid state [4]. Furthermore, local disorder can also induce localized amorphization even in crystalline phase [16]. According to the simple and early-established theoretical considerations, the transition between the amorphous and the crystalline states, such as the “umbrella flip” model [17], has been attributed to rapid crystallization from the intrinsic similarity in atomic arrangements. However, their atomic arrangements are not yet clear, which results in poor understanding of the phase exchange mechanism. The evidence observed experimentally also indicates that the local atomic arrangements in the amorphous and crystalline states differ considerably [18]. In addition, several recent theoretical reports strongly suggest that the “umbrella flip” model needs to be reevaluated [1,4,19,20].

To further fully understand the underlying physics behind phase change, a solid understanding of the bonding mechanism is a prerequisite. Thus, many studies have been conducted to reveal the chemical bonding nature of phase change materials [20,21,22]. For example, Wuttig et al. found that resonant bonding is a unique fingerprint and is responsible for the physical properties of the crystalline phase change materials [21]. Through electron localization function analysis, Ma et al. identified the chemically bonded atom pairs and found that the threefold p-type bonding is prominent in amorphous Ge_2_Sb_2_Te_5_ [20]. Recent publications by Kolobov et al. also confirmed the proposed resonant bonding [7] and p-type bonding [23] in GeTe.

Directly knowing the force acting between atoms in the phase change material can help us understand the atomic origin of the rapid and reversible switch between the crystalline and amorphous phases, because the force constants (FCs) can quantitatively show us the binding strengths between specific atoms. These strengths are, however, very difficult to be determined experimentally, due to the extremely small distance between atoms in nature. When the eigenvectors are not known, things will get worse because there is no unique solution if one wants to extract the FCs from the measured phonon frequencies [24,25]. In the present study, to gain understanding of the origin of phase change mechanisms, we have extracted the FCs between the atoms in GeTe and Sb_2_Te_3_ through the approach of chemical bonding analysis by using the first-principles calculations.

## 2. Materials and Methods

Theoretically, the FCs between atoms can be calculated directly by combining the frozen-phonon method with the first-principles calculations [26,27]. The phonon frequencies can be reduced using the Fourier transformation of the FCs. The reliability of these obtained FCs can then be verified through the comparison between the deduced phonon frequencies from the calculated FCs and the available Raman, infrared, and neutron diffraction experimental data. Without loss of the generality, stable GeTe and Sb_2_Te_3_ phases are studied in the present work. The reason why the GeTe-Sb_2_Te_3_ pseudobinary compounds are not chosen is that the precise atomic structures of these pseudobinary compounds remain unknown, due to the randomly distributed Ge and Sb atoms and the intrinsic vacancies [28]. For example, a recently proposed atomic model for Ge_2_Sb_2_Te_5_ contains 240 atoms in the unit cell [19], which will become a very challenging problem in terms of computing demands.

In the present first-principles calculations, the Vienna ab initio simulation package (VASP) [29,30] and ALKEMIE (an intelligent computational platform for accelerating materials discovery and design) [31] are used. The calculations are conducted in a plane-wave basis with cut-off energy of 400 eV. The projector-augmented-wave potentials are used to describe the electron-ion interaction. The electron configuration is [Ar]3d^10^4s^2^4p^2^ for Ge, [Ar]5S^2^5P^3^ for Sb, and [Ar]5S^2^5P^4^ for Te [32]. The exchange and correlation effects are described by the generalized gradient corrections proposed by Perdew-Burke-Ernzerhof [33]. The integration in the Brillouin zone is performed on the special *k* points determined from the Monkhorst-Pack [34] scheme over 17 × 17 × 17 and 13 × 13 × 13 meshes for GeTe and Sb_2_Te_3_, respectively. The unit cells are fully relaxed with respect to both their volume and shape, as well as to the atomic positions.

Furthermore, the FC matrix and the corresponding phonon frequencies are calculated with the frozen-phonon method, which is implemented in the alloy theoretic automated toolkit (ATAT) [35,36], combined with the VASP. The cutoff distance in constructing a supercell for all the FC calculations is 20 Å, resulting in 256 and 208 atoms for GeTe and Sb_2_Te_3_, respectively. Gamma point is used in the integration of the Brillouin zone. A test with 3 × 3 × 3 Monkhorst-Pack mesh for Sb_2_Te_3_ with 208 atoms shows that there is almost no difference between the results integrated from the 3 × 3 × 3 mesh and gamma point. In addition, opposite-sign perturbations are also applied to ensure that the effect of the third-order FCs cancels out exactly in the fit [36].

To study the distortion effect, a 4 × 4 × 1 supercell containing 96 Sb and 144 Te atoms constructed from the conventional hexagonal cell of Sb_2_Te_3_ is introduced in the present molecular dynamics (MD) simulations. Considering that the short simulation time can be approached with the current first-principles calculations, the simulation system is considered as in an isolated thermodynamic system, i.e., the microcanonical ensemble (NVE) is used in the present MD simulations. An initial temperature is set to 300 K. Each MD job is run to 3 ps with 3 fs time step. Results collected from two selected MD jobs running to 12 ps show that there are no qualitative differences between the results due to short running time.

## 3. Results

We have carried out detailed calculations of phonons in GeTe and Sb_2_Te_3_. The crystalline structures used in the calculations are shown in this section. The calculated phonon frequencies at the gamma point are listed for GeTe and Sb_2_Te_3_, respectively. The selected phonon spectra and phonon density of states along with the available experimental data are alsp shown. The FCs are then reported, while the electronic origins of the unusual FCs are demonstrated. Next, the important findings in these results are given in detail.

### 3.1. Crystalline Structures

**Figure 1:**
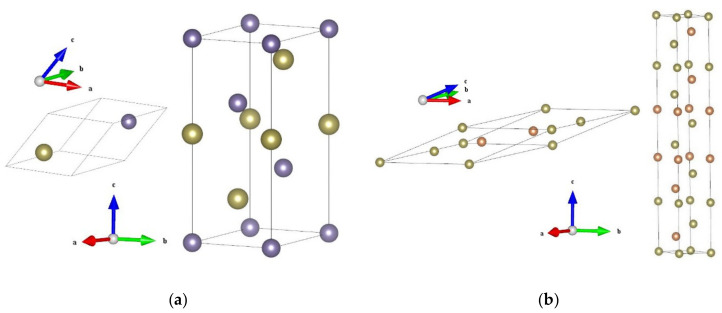
Primitive (left) and conventional (right) cells of (**a**) GeTe and (**b**) Sb_2_Te_3_. The purple, orange and brown balls represent Ge, Sb and Te atoms, respectively.

GeTe is crystallized in the rocksalt structure at temperatures above ~700 K, and will distort into a rhombohedral structure at lower temperatures [28]. Sb_2_Te_3_ crystallizes in a rhombohedral structure as well [28]. The primitive cells of these two stable phases are shown in Figure 1, with the hexagonal conventional cells. The simulated lattice constant of the GeTe primitive rhombohedral cell containing two atoms is 4.3431 Å with the interaxial angle of 58.089°, which agrees well with the corresponding experimental values of 4.3061 Å and 57.942° [37], respectively. The simulated lattice constant of the Sb_2_Te_3_ primitive rhombohedral cell containing five atoms is 10.5759 Å with the interaxial angle of 23.706°, which is slightly larger than the corresponding experimental values of 10.4469 Å and 23.551° [38].

### 3.2. Phonon Frequencies

**Figure 2:**
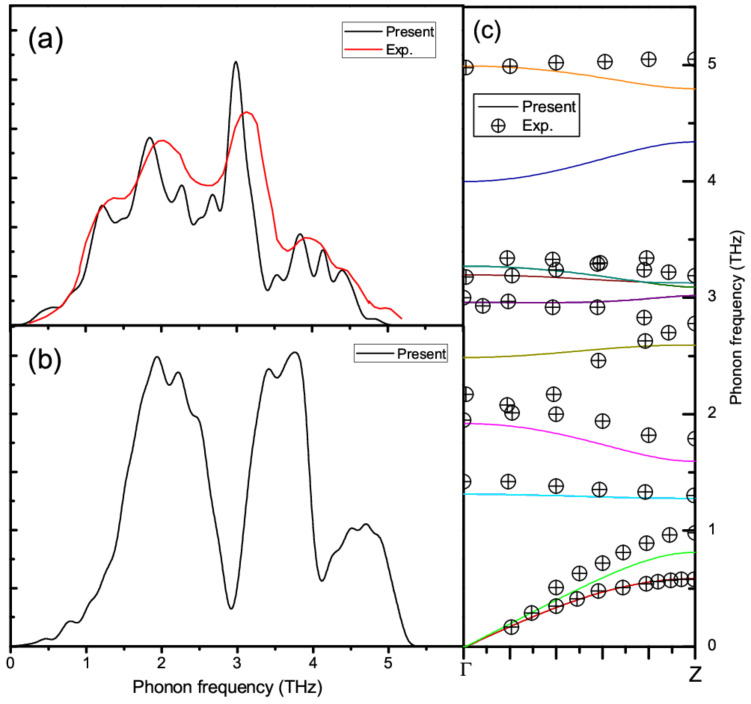
(**a**) Phonon DOS (black line) and the time-of-flight spectrometer data [46] (red line) for Sb_2_Te_3_, (**b**) phonon DOS for GeTe and (**c**) phonon spectra for Sb_2_Te_3_ with neutron scattering data [45] (cross-filling of circle).

Table 1 and Table 2 list the calculated phonon frequencies at the Brillouin zone center (in cm^−1^) for GeTe and Sb_2_Te_3_, respectively, as well as the available experimental data and other theoretical results [39,40,41,42,43,44]. Figure 2a–c show the phonon density of states (DOS) for Sb_2_Te_3_ and GeTe, and phonon spectra for Sb_2_Te_3_ along Γ-Z directions in reciprocal space, respectively, as well as the available neutron scattering [45] and time-of-flight spectrometer [46] data. Here, group theoretical analyses show that in rhombohedral GeTe with two atoms in unit cell, with E mode it is double degenerate, two modes A1 and E are Raman and infrared active, respectively [41]. In addition, for Sb_2_Te_3_, there are five atoms in unit cell, thus total 12 optical modes exist. Due to the fact that E and A modes represent displacement in the a–b plane and along the c axis, respectively, all the four vibration E optical modes along Γ–Z direction are double degenerate; thus, only eight frequencies can be measured with Raman observation [47]. In short, the overall good agreement between the calculated phonon frequencies and the measured data imply that the FCs calculated from the supercells are reasonable.

### 3.3. Force Constants

**Figure 3:**
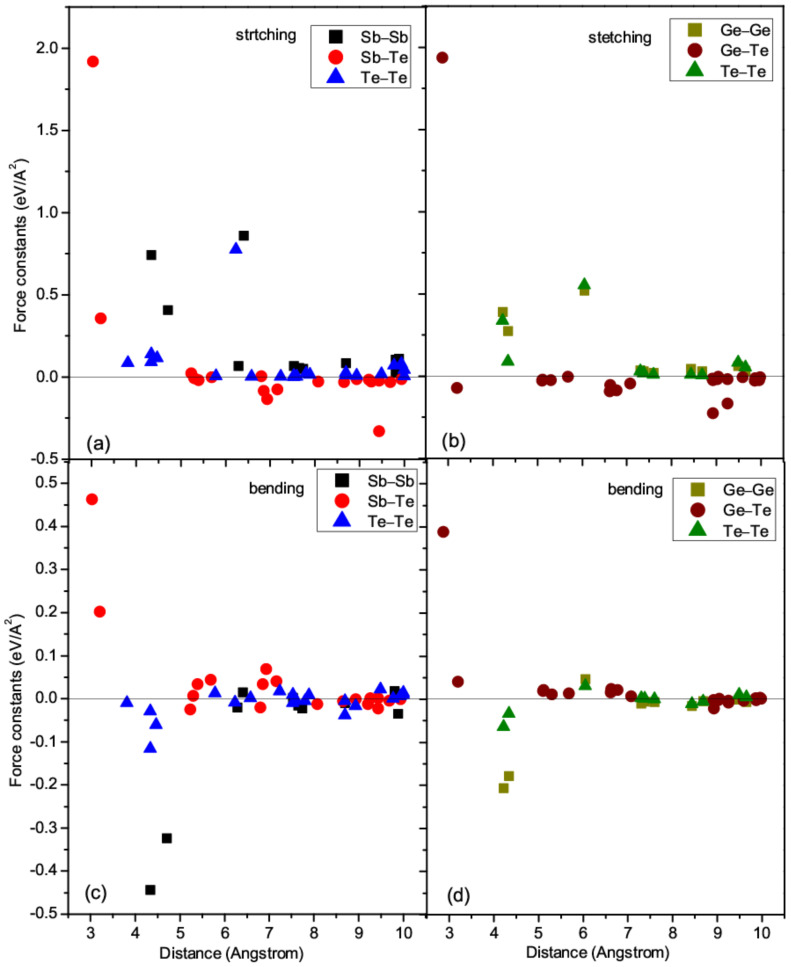
Stretching FCs of (**a**) Sb_2_Te_3_ and (**b**) GeTe, and bending FCs of (**c**) Sb_2_Te_3_ and (**d**) GeTe.

Figure 3 demonstrates the calculated stretching and bending FCs in crystalline GeTe and Sb_2_Te_3_. The stretching action is defined as a change in the length of the bond between two atoms due to an axial force, while the bending action refers to a change in the lateral distance between two atoms due to a force normal to the bond direction. Technically, the stretching FCs can be calculated by:

(1) $$Stretching\;FC={\vec{u}}^{T}\cdot (fc:\vec{u})$$

where $\vec{u}$ is the 3 × 1 unit vector defined as the direction from atom a to atom b, ${\vec{u}}^{T}$ is the transposition vector of $\vec{u}$ and fc is the 3 × 3 FC matrix between atom a and atom b. Bending FCs are the average of the two perpendicular bending FCs, which can be calculated by:

(2) $$B1={\vec{a}}^{T}\cdot (fc:\vec{a})$$

and

(3) $$B2={\vec{b}}^{T}\cdot (fc:\vec{b})$$

where unit vector $\vec{a}$ is perpendicular to vectors $\vec{u}$ and $\vec{b}=\vec{a}⊗\vec{u}$, normal to the plane containing $\vec{u}$ and $\vec{a}$. *B1* and *B2* represent the separated bending FCs along the two perpendicular directions. It needs to be noted that this stretching-bending force constant model was first proposed by Ceder et al. [50]. In this model, the coordinate system of each force constant matrix is transformed following Equations (1), (2) or (3). Thus, the FCs shown in Figure 3 are not the original 3 × 3 FC matrix but a scalar.

Some general features can be found from Figure 3. The first and most important unusual feature is that the stretching FCs of the second nearest-neighbor (NN) Sb-Te and Ge-Te bonds are 0.372 and −0.085 eV/Å^2^, respectively, much lower than those of the first NN bonds, which are equal to 1.933 eV/Å^2^. Besides a rather small bending FC (<0.05 eV/Å^2^), the stretching FC of the Ge-Te bond at the second NN distance even becomes negative. These dramatic changes of the FCs are really astonishing, as the differences between the first and second NN distances are only 0.17 Å and 0.33 Å for Sb_2_Te_3_ and GeTe, respectively.

The second interesting feature is that the bending FCs of the Ge-Ge and Sb-Sb bonds at the first and second NN distances between like atoms show large negative values, suggesting that the Ge-Ge and Sb-Sb bonds would be ready to move under slight shear perturbations, in accordance with their layer structure nature. It needs to be noted that negative FC between two atoms along a certain direction does not mean that the two atoms will leave, for the stability of a structure depends on the collective behavior of atomic vibrations [51,52]. As the phonon DOS shown in Figure 2a,b, all phonon modes are really positive, which means the two structures are dynamically stable. More details can be found from the separated bending FCs. In GeTe, the bending FCs of Ge-Ge bonds at a distance of 4.2170 Å are *B1* = −0.170 eV/Å^2^ and *B2* = −0.242 eV/Å^2^, while these values become *B1* = −0.014 eV/Å^2^ and *B2* = −0.342 eV/Å^2^ at a distance of 4.3431 Å. The bending FCs of these slightly longer Ge-Ge bonds are rather asymmetric, suggesting that the shear deformation would be strongly direction-dependent. In Sb_2_Te_3_, the Se-Se bending FCs are *B1* = −0.309 eV/Å^2^ and *B2* = −0.577 eV/Å^2^ at a distance of 4.3447 Å, while these values become *B1* = −0.046 eV/Å^2^ and *B2* = −0.602 eV/Å^2^ at a distance of 4.7076 Å. More profound asymmetric shear behaviors are observed.

Thirdly, the second largest stretching FCs are found at the third NN distance between like atoms (around 6 Å), higher than most of the shorter bonds (except for the bonds between the first NN unlike atoms), making significant contribution to the stability of the phase. Much shorter bonds such as Ge-Ge and Sb-Sb at around 4 Å, and Sb-Te and Ge-Te at around 3 Å, are weaker than these longer bonds between like atoms.

Fourthly, the overall distributions of the stretching and bending FCs are similar for GeTe and Sb_2_Te_3_. The highest stretching and bending FCs are around 2.0 eV/Å^2^ and 0.4 eV/Å^2^, respectively, for both GeTe and Sb_2_Te_3_. This similarity is consistent with the similar rhombohedral symmetries of these two stable phases.

### 3.4. Origin of the Unusual Force Constants

**Figure 4:**
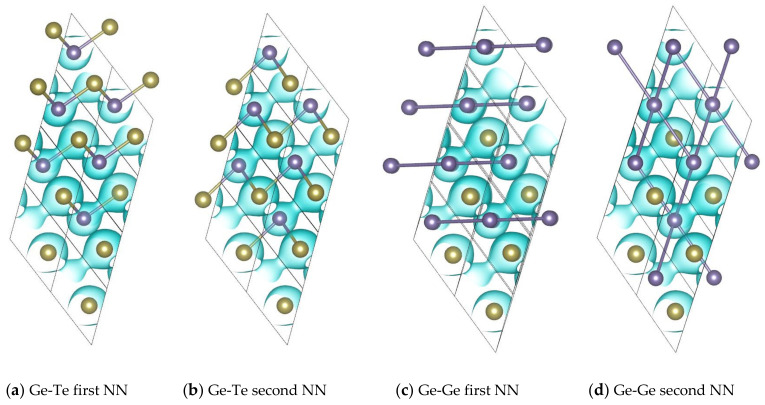
Crystal structures shown with the isosurface at −0.035 electrons per bohr^3^ in spatial valence charge density and the noteworthy bonds for (**a**) Ge-Te first NN, (**b**) Ge-Te second NN, (**c**) Ge-Ge first NN, and (**d**) Ge-Ge second NN. The bonds between atoms are shown with bicolor cylinder type.

It is of high scientific interest to find out the physical origin of these unusual FCs. Hence, in the next section, the spatial valence charge density is analyzed to reveal the bonding nature in the studied structures [53]. Figure 4 shows the crystal structures of Ge-Te and Ge-Ge bonds at the first and second NN distances between unlike atoms and between like atoms, respectively, as well as the isosurface with value of −0.035 number of electrons per bohr^3^ in spatial valence charge. From the comparison between Figure 4a,b, one can clearly see that higher charge densities are distributed along the first NN Ge-Te bonds, but are much lower than those along the second NN Ge-Te bonds. These differences suggest that the binding of the first NN Ge-Te is covalent type and much stronger than the binding of the non-covalent second NN bonds, which leads to the large difference between the FCs of these two bonds. Figure 4c shows that the first NN Ge-Ge bond is entirely located in the Ge layer, hence the tensile or compressive movement is limited by the adjacent Ge atoms, resulting in positive stretching FCs. In the out-of-plane direction, however, there are three equally distributed strong covalent first NN Ge-Te bonds, which are oriented by 42.7° to the Ge layer, exerting the attractive force to draw Ge atoms out from the Ge layer, and thus resulting in large negative bending FCs. The situation is similar for the second NN Ge-Ge bonds as shown in Figure 4d. The only difference is that the covalent Ge-Te bonds are not equally distributed, resulting in large asymmetric bending FCs. The reason why the second largest FCs of Ge-Ge and Te-Te bonds occur at distances around 6 Å is that their characteristics are similar to those of the first and second NN Ge-Te bonds. Hence, they are covalent bonds to some extent.

**Figure 5:**
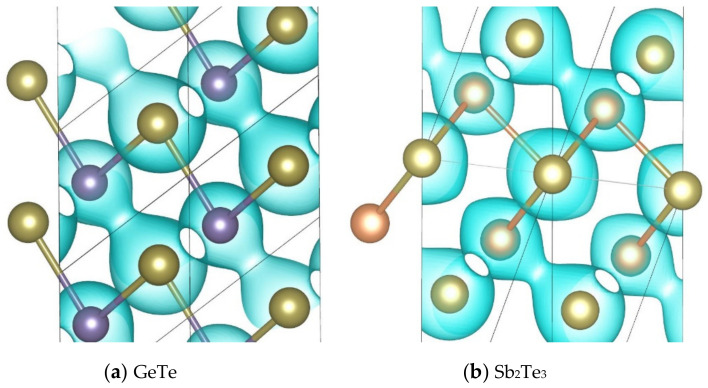
The bonds at the second NN between unlike atoms with the isosurface at −0.035 electrons per bohr^3^ in the spatial valence charge density of (**a**) GeTe and (**b**) Sb_2_Te_3_.

Due to the similar rhombohedra symmetry, the situations for Sb_2_Te_3_ are not discussed in detail. A noteworthy difference as demonstrated in Figure 5b is that although the isosurfaces near the second NN Sb-Te are not as profound as those near the first NN Sb-Te bonds, they are not spherical but bulged, indicating some covalent binding characteristics. These characteristics cause the stretching and bending FCs of the second NN Sb-Te bonds to be positive, different from those of the second NN Ge-Te bonds (shown in Figure 5a), where the stretching FC is negative and the bending FC is close to zero.

## 4. Discussion

A thorough investigation on these unusual FCs will certainly help us understand the mechanism of phase change. From the calculated FCs, we can conclude that the first NN Ge-Te and Sb-Te bonds have much larger FCs than the other bonds. With this insight, we can explain a lot of experimental observations and simulation results for phase change materials. For example, through atomic simulations, Caravati et al. [54] found that the sharp peak of the bond angle distribution around Sb or Te atoms in amorphous Sb_2_Te_3_ is centered at 90.46°. According to our structure relaxation of Sb_2_Te_3_, the dihedral angle between the adjacent first NN Sb-Te bonds is 91.38°, while the dihedral angle between the adjacent second NN Sb-Te bonds is 85.36°. Since the first NN Sb-Te bonds have much larger FCs than the other bonds in Sb_2_Te_3_, the reported angle distribution by Caravati et al. is very close to that of the first NN Sb-Te bonds. This situation also holds true for GeTe-Sb_2_Te_3_ pseudobinary compounds such as Ge_2_Sb_2_Te_5_. From the structure relaxation of this study, the dihedral angle between the adjacent first NN Ge-Te bonds is 94.6°, while that of the adjacent second NN Ge-Te bond is 82.39° in GeTe. Through atomic simulations, Sun et al. [55] found that the sharp peaks of the bond angle distributions around Ge, Sb and Te atoms in amorphous Ge_2_Sb_2_Te_5_ are centered at ~97°, ~90° and ~89°, respectively. That is to say, although the total pair correlation functions for amorphous and crystalline states are rather different [18], microscopically the strongest Te-Ge and Te-Sb binding is preserved in phase change materials. Furthermore, other examples can also be seen in rather different situations. We all know that besides temperature, high pressure can also induce phase change. According to Krbal et al. [56], among the remaining bonds in GeSb_2_Te_4_ under pressure of 46 GPa, 59% of them are Ge-Te and Sb-Te bonds, while only 8% of them are Ge-Ge and Sb-Sb bonds.

**Figure 6:**
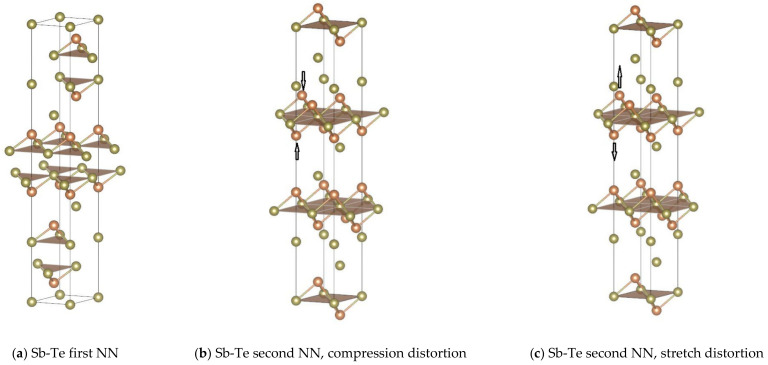
The first and second NN Sb-Te interactions shown in conventional hexagonal cell with polyhedral view: (**a**) first NN, (**b**) second NN, compression distortion, and (**c**) second NN, stretch distortion with arrows.

We would like to further stress the importance of quantitatively knowing the FCs. Recently, Kolobov et al. [4] demonstrated that appropriate distortion in crystals can trigger the destruction of the subsystem with weaker bonds and the subsequent collapse of the long-range order, which will generate the amorphous phase without going through the liquid state, thus making it possible to significantly reduce the energy consumption through the use of shorter pulses or excitation of coherent optical phonons. As motivated by Kolobov et al. [4], we further demonstrate that through locally distorting the bonds, the phase change materials can be amorphized through MD simulations. From the results of FCs in Sb_2_Te_3_, we know that the FCs at the second NN distance are much lower than those at the first NN distance between unlike atoms, although the differences between the first and second distances are only 0.17 Å. Figure 6 shows the first and second Sb-Te interactions in hexagonal conventional cell with polyhedral view. From the figure, it is noted that each Sb atom is connected with three surrounding Te atoms to form a tetrahedron. However, it is not possible to entirely separate the first and second NN interactions between Sb-Te atoms. Nevertheless, we consider here one possible distortion method, which is to compress or stretch the two Sb atoms along the <001> direction as shown in Figure 6b,c, in order to weaken or strengthen, respectively, the first and second interactions simultaneously.

**Figure 7:**
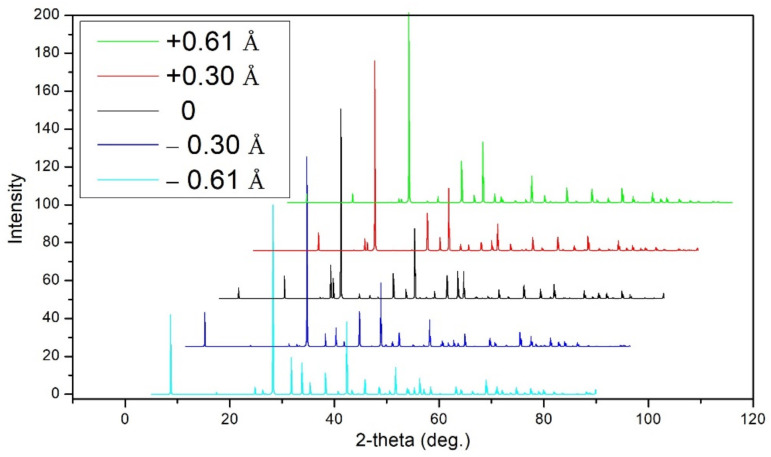
X-ray diffraction spectra of Sb_2_Te_3_ generated for the ideal and distorted structures.

A 4 × 4 × 1 supercell containing 240 atoms constructed from the conventional hexagonal cell of Sb_2_Te_3_ is introduced to study the above mentioned distortion effect. The step size of the distortion along the <001> direction is 0.5% of the lattice constant of the conventional hexagonal cell in the direction normal to the basal plane. For the purpose of convenience, we use the negative value to represent the elongation distortion of the Sb atoms along the <001> direction in the tetrahedron, as well as the positive value to represent the compressive distortion. The distorted structures still retain the long-range order of the crystalline phase, as confirmed by the generated X-ray diffraction pattern shown in Figure 7. In the X-ray simulation, the wavelength λ = 1.540562 Å in Cu Kα1 emission lines is used as radiation source. The diffraction occurs in the range from 5 to 90° with an increment size of 0.05°. In all distorted structures, the spectra are dominated by the crystalline peaks, showing characteristics of the ideal Sb_2_Te_3_ model. These observations suggest that the introduced disturbances are local in nature, i.e., the structure remains crystalline evidenced by the Bragg X-ray diffraction experiment.

**Figure 8:**
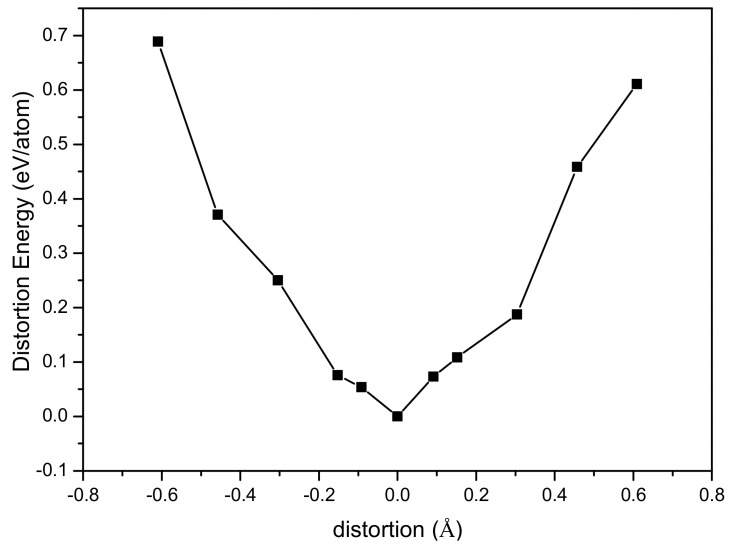
The distortion energies of Sb_2_Te_3_ vary with the distortion distances along the <001> direction.

From the system energy point of view, the distortion energy varies with the distortion distance along the <001> direction as shown in Figure 8. Bond elongation along the <001> direction of the tetrahedron results in the compression of the first NN Sb-Te bonds which features strong force constants, while the tension of the second NN Sb-Te bonds features weak force constants. From Figure 8 it is noted that when the magnitude of distortion is smaller than 0.17 Å, the distortion energy of stretching process is higher than that of compressing process, suggesting that the strong bonds are more difficult to be compressed and the weak bonds are easier to be stretched, consistent with the FCs results. When the magnitude of distortion is larger than 0.17 Å, the bond initially featuring strong FCs will become longer than the bond initially featuring weak FCs, leading to the exchange of the relative strong and weak characteristics of FCs, namely, the initial stronger bonds become weaker and the initial weaker bonds become stronger.

**Figure 9:**
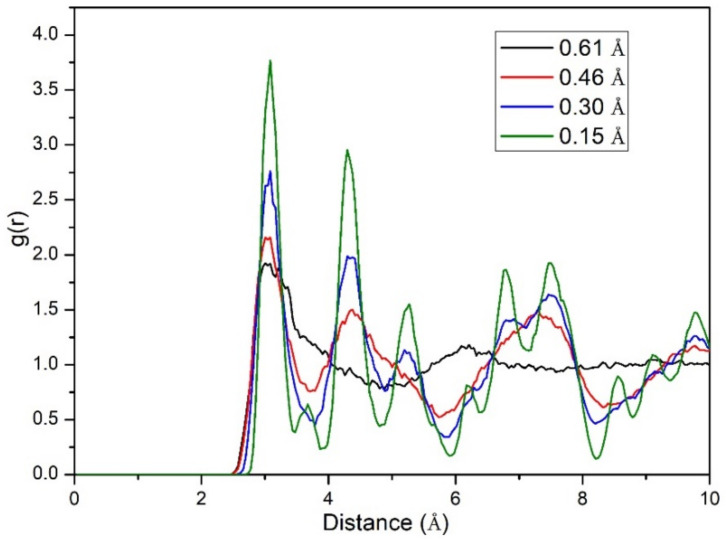
RDF of different distorted structures of Sb_2_Te_3_ after 3ps NVE MD simulations. It is clearly shown that when the distortion is large enough, the locally distorted structure will lose its long-range order and be finally amorphized.

The distorted structures are then deployed into MD simulations to see whether these locally disturbed crystalline structures can become amorphous without initially presetting very high temperature in MD simulations [57]. The obtained pair correlation function, or radial distribution function (RDF) g(r) for different distortions is shown in Figure 9. To simplify the figure, the average RDF values with the equal distance of the stretching and compressing distortion are shown here. From the figure, one can find that with increasing distortion, the initial crystalline structure will finally become amorphous during the simulated time. As different distortion will lead to different distortion energies, quantitatively we can know the different bond strengths between atoms. This finding is of importance for guiding us on where and how the appropriate distortion should be applied, and thus optimizing the design and fabrication of components/devices based on phase change materials with lower energy consumption.

Moreover, it is worthy to note that for GeTe and Sb2Te3, rhombohedral structure is closely related to rocksalt structure. For example, GeTe will distort into a rhombohedral structure at lower temperatures from rocksalt structure at temperatures below ~700 K [28]. Is the direction of the vector connected with the direction of the atomic displacement in the phase-shift from rhombohedral structure to rocksalt structure or vice versa? Do the calculated negative FCs of the rhombohedral GeTe reflect, in a relevant way, the distorted rocksalt cubic structure? The present work concentrates on the transition from the crystalline to the amorphous state at room temperature; such investigations are beyond the scope of this paper and will be the subject of future study. Transformation between rhombohedral structure and rocksalt structure from other perspectives can be found in literature as [28,47,58].

## 5. Conclusions

Through analyzing the FCs of GeTe and Sb_2_Te_3_ by using the first-principles calculations, we find that the overall characteristics of the stretching and bending FCs are similar for GeTe and Sb_2_Te_3_. In particular, (1) the stretching FCs between unlike atoms at the first NN distance in GeTe and Sb_2_Te_3_ are equal to 1.933 eV/Å^2^; (2) the stretching FC of Sb-Te bonds at the second NN distance is only 19% of that at the first NN distance, while the stretching FC of Ge-Te bond at the second NN distance even becomes negative; (3) the bending FCs of the first and second NN bonds between like atoms feature large negative values, and are strongly direction-dependent; and (4) the second NN bonds between like atoms in Sb_2_Te_3_ and GeTe are found to have the second largest stretching FCs.

The origin of these unusual FCs is explained through analyzing the spatial valence charge density. Our first-principles molecular dynamic simulations reveal the possible amorphization of phase change materials through local distortions of the bonds featuring weak or strong FCs, while the crystalline structure remains observed by the X-ray diffraction experiment. We believe that the obtained FCs are of a general nature and thus can be used to understand the phase change mechanism of the GeTe-Sb_2_Te_3_ pseudobinary compounds. This interesting result is of importance for understanding phase change materials as it might provide a recipe to generate two-phase-based devices with lower energy consumption.

## Figures and Tables

**Table 1:** Calculated phonon frequencies (in cm^−1^) from the present and previous studies at the zone center for GeTe, as well as the available Raman experimental data.

Method	VASP	Cal.	Cal. ^a^	Cal. ^b^	Exp.	Exp.	Exp.
E(TO)	90.63	73	73	73	98	80	88
A1(LO)	123.94	152	121	115	140	122	123
Ref.	Present	[39]	[39]	[40]	[41]	[48]	[40]

^a^ Complete screening of the dipole-dipole interaction; ^b^ Consideration of hole (hole concentration is 2.1 × 10^21^ holes/cm^3^).

**Table 2:** Calculated phonon frequencies (in cm^−1^) from the present and previous studies at the zone center for Sb_2_Te_3_, as well as the available experimental data.

Eg1	A1g1	Eu1	Eu2	A1u1	Eg2	A1u2	A1g2	Method	Ref.
43.7	64.0	82.9	98.7	106.6	109.1	133.4	166.5	VASP	Present
46	62	72	99	108	113	145	166	PWSCF	[44]
	69				112		165	Exp.	[42]
43		56						Exp.	[43]
		67	91	110		157		Exp.	[49]

PWSCF: Plane-Wave Self-Consistent Field software.

## Data Availability

Data are contained within the article.
